# The effects of information-seeking behaviours on prevention behaviours during the COVID-19 pandemic: the mediating effects of anxiety and fear in Korea

**DOI:** 10.4178/epih.e2021085

**Published:** 2021-10-19

**Authors:** Kwanghyun Kim, Jisu Yang, Ye Jin Jeon, Yu Jin Lee, Youngrong Lee, Hyeon Chang Kim, Karestan Koenen, Yong-Chan Kim, Sun Jae Jung

**Affiliations:** 1 Department of Preventive Medicine, Yonsei University College of Medicine, Seoul, Korea; 2 Department of Public Health, Yonsei University Graduate School, Seoul, Korea; 3 Department of Epidemiology, Harvard University T. H. Chan School of Public Health, Boston, MA, USA; 4 College of Communication, Yonsei University, Seoul, Korea

**Keywords:** COVID-19, Information seeking behaviour, Health behaviour, Fear, Anxiety

## Abstract

**OBJECTIVES:**

Identifying determinants of prevention behaviours during the emergence of a new infectious disease is important. We investigated the associations between information-seeking and prevention behaviours during the coronavirus disease 2019 (COVID-19) pandemic and mediating effects of psychiatric factors.

**METHODS:**

In total, 1,970 participants from the Cardiovascular and Metabolic Etiology Research Center cohort participated in an online survey 55 days after the first COVID-19 case in Korea was diagnosed. Time spent seeking information related to COVID-19; information sources; psychiatric factors, including anxiety, depression, post-traumatic stress symptoms (PTSS), and the fear of COVID-19; and prevention behaviours were examined. The mediating effect of psychiatric factors was estimated using mediation analysis.

**RESULTS:**

Time spent seeking information and information sources affected several behavioural responses. In men, anxiety mediated associations between information-seeking and prevention behaviours, including purchasing sanitary supplies (effect size [ES], 0.038; 95% confidence interval [CI], 0.002 to 0.095) and hoarding (ES, 0.029; 95% CI, 0.002 to 0.068). The fear of COVID-19 also mediated associations between information-seeking and prevention behaviours including refraining from going out (men: ES, 0.034; 95% CI, 0.009 to 0.068; women: ES, 0.052; 95% CI, 0.030 to 0.080), wearing face masks (men: ES, 0.085; 95% CI, 0.031 to 0.184), avoiding public transportation (men: ES, 0.020; 95% CI, 0.000 to 0.044; women: ES, 0.031; 95% CI, 0.015 to 0.051), hoarding (women: ES, 0.051; 95% CI, 0.029 to 0.792), and trying alternative remedies (men: ES, 0.024; 95% CI, 0.004 to 0.053). Depressive symptoms and PTSS did not have any mediating effects.

**CONCLUSIONS:**

While the availability of information related to COVID-19 can help prevent infections, it can also promote anxiety and fear, leading to negative behaviours such as hoarding and trying unverified alternative treatments.

## INTRODUCTION

Since the confirmation of the first case of coronavirus disease 2019 (COVID-19) in Wuhan, China [1], COVID-19 has spread worldwide, infecting as many as 250 million individuals in more than 180 countries [2]. Although pharmaceutical treatments for COVID-19 are being developed and vaccination is in progress, the effects of vaccination against newly emerging severe acute respiratory syndrome coronavirus-2 variants have not yet been investigated [3]. Therefore, non-pharmaceutical interventions, such as social distancing [4] and wearing face masks [5], are still of utmost importance as prevention strategies. Since the willingness of the public to comply with prevention measures recommended by public health officials is known to be a crucial aspect of infection control [6], it is important to understand the nature of public compliance with prevention measures and determinants that affect behavioural patterns among the general public.

Few studies have examined the determinants of behavioural adaptations during the COVID-19 pandemic. During the 2009/2010 influenza A (H1N1) pandemic, a study conducted in Germany showed that those who obtained information from conventional media, including radio, television, and newspapers, were less compliant concerning vaccination, while those informed by physicians or official authorities were more compliant [7]. In addition, a study conducted among 997 British adults found that information about a disease and its perceived severity can affect public behavioural patterns and found that that implementation of recommended prevention measures was associated with both the perceived sufficiency of pandemic-related information (odds ratio [OR], 1.3, 95% confidence interval [CI], 1.1 to 1.5) and the perceived severity of the disease (OR, 1.6; 95% CI, 1.4 to 1.8) [8].

Links between information-seeking behaviours and health anxiety have been identified in previous studies [9]. During an emerging pandemic, a large proportion of excess information is often not supported by evidence and can be contradictory [10,11]. As a result, unlike normal public health circumstances during which health information decreases uncertainty and anxiety, contradictory information and a lack of significant evidence for information can exacerbate distress and risk perceptions during a pandemic [12,13]. As a result, seeking health information during a pandemic can result in increased anxiety [14], post-traumatic emotional responses [15], and depressive symptoms [16]. An increase in information-seeking behaviours and subsequent psychological symptoms during the ongoing COVID-19 pandemic, which the World Health Organization has referred to as an ‘infodemic’ [17], has been found to be associated with anxiety and post-traumatic stress symptoms (PTSS) [14,15].

Since public anxiety and fear are important determinants of behavioural responses that can facilitate or prevent effective prevention strategies, their effects on prevention strategies should be examined in the context of the COVID-19 pandemic. In an earlier example, an online survey of Dutch adults found that anxiety during the influenza A (H1N1) pandemic increased respondents’ intentions to comply with government-advised preventive measures (OR, 2.81; 95% CI, 2.14 to 3.70) [18]. A recent study that examined the ongoing COVID-19 pandemic also found that concerns about COVID-19 affected prevention behaviours among the public, although the specific effects of general anxiety on COVID-19-related behaviours are still unknown [19].

**Figure 1.**
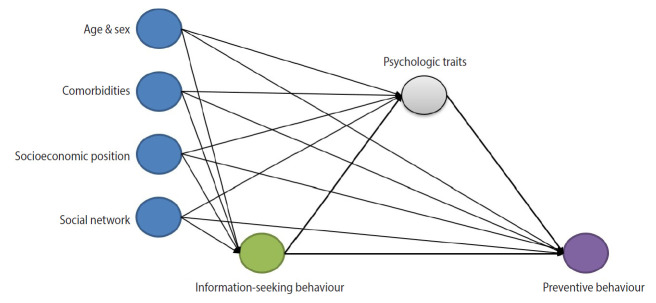
Directed acyclic graph depicting associations among information-seeking behaviours, psychologic symptoms, and preventive behaviours.

Since general anxiety and fears related to specific situations differ in nature and result in different psychological actions [20], we investigated the mediating effects of these psychological changes separately to determine the degrees to which the psychological effects of information-seeking behaviours ultimately result in changes in compliance with prevention behaviours. Therefore, we aimed to examine the effects of information-seeking behaviours on prevention behaviours and separately investigate the mediating effects of general anxiety and the fear of COVID-19 (Figure 1).

## MATERIALS AND METHODS

### Study participants

The Cardiovascular and Metabolic Etiology Research Center study is an ongoing community-based multicentre prospective cohort study designed to investigate the aetiology of cardiovascular and metabolic diseases in Korea [21]. During the baseline examination, which was conducted between 2013 and 2018, the demographic information, anthropometric measures, past medical histories, and psychiatric traits of the participants were assessed, and biochemical analyses of blood and urine samples from the participants were conducted.

**Figure 2.**
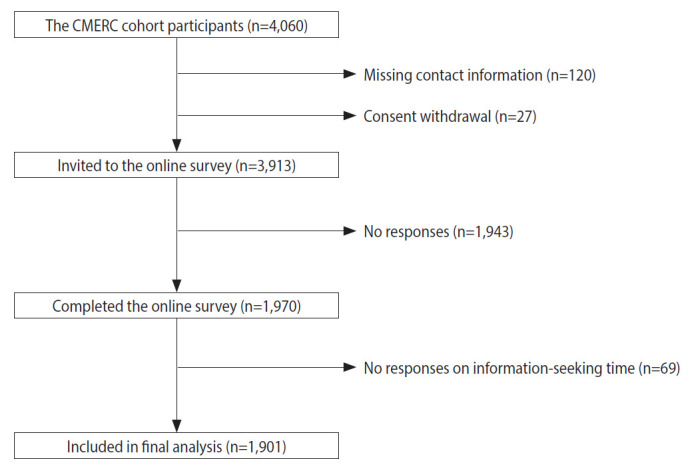
Flow chart of participant enrollment and study design from Cardiovascular and Metabolic Disease Etiology Research Center (CMERC) cohort.

For this study, we contacted participants from the community-based population recruited by the Yonsei University College of Medicine (n=4,060), most of whom resided in Seoul and other north-western cities of Korea (Incheon, Goyang, and Gimpo). Postal mail invitations to the online survey for assessing mental health during the COVID-19 pandemic were sent on March 11 and March 12, 2020. Participants without contact information (n=120) and participants who withdrew consent (n=27) were excluded. The remaining 3,913 participants received a link to the online survey via mobile short message service on 2 occasions, on March 14 and March 18, 2020, after which 1,970 responses were gathered (response rate, 50.3%). Participants who did not provide a response concerning their total time spent seeking information (n=69) were excluded, leaving 1,901 participants for the final analysis (Figure 2).

#### Online survey for assessing mental health, information-seeking, and prevention behaviours

An online survey was designed to assess the participants’ information-seeking behaviours, behavioural responses specific to the outbreak of COVID-19, and psychiatric traits. To gather the data on information-seeking behaviours, participants were asked a multiple-choice question on the information sources they used to obtain COVID-19-related information, for which the possible answers were mass media (including Internet newspapers), government organisations (including webpages), medical personnel or institutions (including webpages), posters and leaflets, portal sites and Internet media (including search engines and YouTube), and other non-professional acquaintances. Since most posters and leaflets tend to be distributed by government agencies, we consolidated government organisations and posters and leaflets into a single category. To assess the average amount of time spent per day gathering information on COVID-19, participants were asked, “How many hours do you spend searching information on COVID-19 per day?” [22-25]. Preventive behaviours were determined by asking the participants to select which behaviours they practised from a list of common prevention behaviours, allowing multiple choices. The options were purchasing sanitary supplies, hoarding food and daily necessities, refraining from going out, wearing face masks, avoiding public transportation, visiting a COVID-19 screening centre to receive tests, and trying unvalidated alternative remedies to prevent COVID-19. Space was also provided for the respondents to describe any other preventive measures they undertook that were not listed.

Various psychiatric traits were also assessed. Depressive symptoms were measured via the Patient Health Questionnaire-9 (PHQ-9), with possible scores ranging from 0 to 27. Anxiety was measured using the Generalized Anxiety Disorder-7 (GAD-7) scale, with possible scores ranging from 0 to 21. PTSS from COVID-19 were measured using the Post-Traumatic Stress Disorder Checklist for DSM-5 (PCL-5), with possible scores ranging from 0 to 80. Korean versions of the PHQ-9 [26], GAD-7 [27], and PCL-5 [28] have been validated in previous studies for use with the Korean population. In addition, the perceived fear of COVID-19 was assessed with a single question (“How much do you fear COVID-19 infection and its health consequences?”), which was answered using a 6-point Likert scale ranging from “no fear at all” (0 points) to “very fearful” (5 points) [29-31]. To test the internal validity of this item, we estimated the correlation coefficients of the fear of COVID-19 with the PHQ-9, GAD-7, and PCL-5. The correlation coefficients and their p-values were 0.326 (p<0.001), 0.487 (p<0.001), and 0.436 (p<0.001), respectively, showing moderate correlation with concurrent variables.

### Covariates

The mean monthly household income and education level were assessed during the baseline examination and were selected as covariates to represent socioeconomic status. Education level was divided into 3 subgroups based on the number of years for which the participants attended school, and they were primary or lower (≤ 6 years), secondary (7-12 years), and tertiary or higher (≥ 13 years).

The participants’ histories of hypertension, diabetes mellitus and related complications, ischaemic heart disease and angina pectoris, cerebrovascular accidents, dementia, connective tissue disease, liver disease, chronic kidney disease, and solid or haematologic neoplasms were collected at the time of the baseline examination and were used to make a Charlson comorbidity index estimation [32]. Pre-COVID-19 depressive symptoms were assessed using the validated Korean version of the Beck Depression Inventory-II [33] and were included as a covariate.

Information on alcohol consumption and cigarette smoking were collected at the time of the baseline assessment. For alcohol consumption, the mean alcohol intake was calculated based on benchmarks from the National Institute of Alcohol Abuse and Alcoholism. A weekly intake of more than 14 servings per week for men and more than 7 servings per week for women was classified as heavy drinking [34]. For cigarette smoking, participants were classified as non-smokers, ex-smokers, or current smokers.

The properties of participants’ social networks were measured at the time of the baseline examination by trained interviewers in accordance with the methods used in the National Social Life, Health, and Aging Project [35]. The size of one’s social network was defined as the number of people, including their spouse and up to 5 others, with whom one discusses important matters. Perceived closeness between the participant and each member of their social network was assessed using a 4-point Likert scale, and mean values of perceived closeness were defined as the mean network closeness.

### Statistical analysis

All analyses were performed separately for men and women because of the known gender differences in attitudes and behaviours towards disease prevention measures [18] and the effects on mental health [36] during pandemics. For the descriptive analyses, the participants were classified into 2 groups based on the amount of time spent seeking information, using the median time (80 minutes) as a reference value (≤ 80 and > 80 minutes), and the characteristics of the participants collected at baseline and during the current study were compared between the groups using the t-test and chi-square test. Multivariable logistic regression analysis was conducted to examine the associations between information-seeking patterns and psychiatric traits and prevention behaviours. Effect plots for the amount of time spent seeking information versus prevention behaviours and anxiety versus prevention behaviours were generated to test the associations.

Mediation analyses were conducted using the PROCESS macro for SAS [37]. The effect sizes (ESs), including the direct and indirect effects of time spent seeking information on depression, anxiety, PTSS, and fear of COVID-19, along with their 95% confidence intervals (CIs), were estimated using bootstrapping, with 5,000 simulations performed for each variable.

All models were adjusted for age, education level, drinking status, smoking status, comorbidities, baseline depressive symptoms, size of social network, and mean network closeness. All analyses were conducted using SAS version 9.4 (SAS Institute Inc., Cary, NC, USA).

### Ethics statement

The Institutional Review Board of Yonsei University Health System, Seoul, Korea, approved the study protocols (Baseline evaluation: 4-2013-0661; COVID-19 mental health online survey: Y-2020-0066). Written consent was obtained before both the baseline examination and the online survey. All procedures contributing to this work complied with the ethical standards of the relevant national and institutional committees on human experimentation and the Helsinki Declaration.

## RESULTS

### Participants’ characteristics

Among the 1,901 respondents included in the final analyses, there were 673 men (35.4%) and 1,228 women (64.6%), with a mean age of 50.84 years. The mean±standard deviation ages for men and women were 50.66±9.98 years old and 50.94±8.94 years old, respectively. The mean follow-up time was 4.38±1.32 years. Mass media was the most frequently used information source (94.7%), followed by Internet-based media (66.8%) and governmental organisations (53.6%).

The participants in our study showed high compliance with the prevention behaviours recommended by government officials. Almost all of the respondents replied that they had purchased sanitary supplies (n=1,695, 89.2%) and wore face masks (n=1,824, 95.9%), and most participants refrained from going out (n=1,515, 79.7%) and avoided using public transportation (n=1,039, 54.7%). Some participants undertook prevention behaviours not recommended by public health officials, such as hoarding food and daily necessities (n=375, 19.7%) and trying unvalidated alternative remedies to prevent COVID-19 (n=683, 35.9%). Positive associations between time spent seeking information and recommended and non-recommended prevention behaviours are summarized in Table 1.

### Associations between information-seeking behaviours, psychiatric traits, and prevention behaviours

Information-seeking behaviours were associated with prevention behaviours, although the associations differed by gender. Men who spent more time seeking COVID-19-related information were more likely to hoard food supplies (OR per hour, 1.11; 95% CI, 1.01 to 1.21). Among the women, positive associations were found for refraining from going out (OR per hour, 1.12; 95% CI, 1.01 to 1.24), avoiding public transportation (OR per hour, 1.06; 95% CI, 1.00 to 1.17), hoarding food supplies (OR per hour, 1.07; 95% CI, 1.00 to 1.13), and trying alternative treatments (OR per hour, 1.18; 95% CI, 1.03 to 1.35) (Table 2).

**Figure 3.**
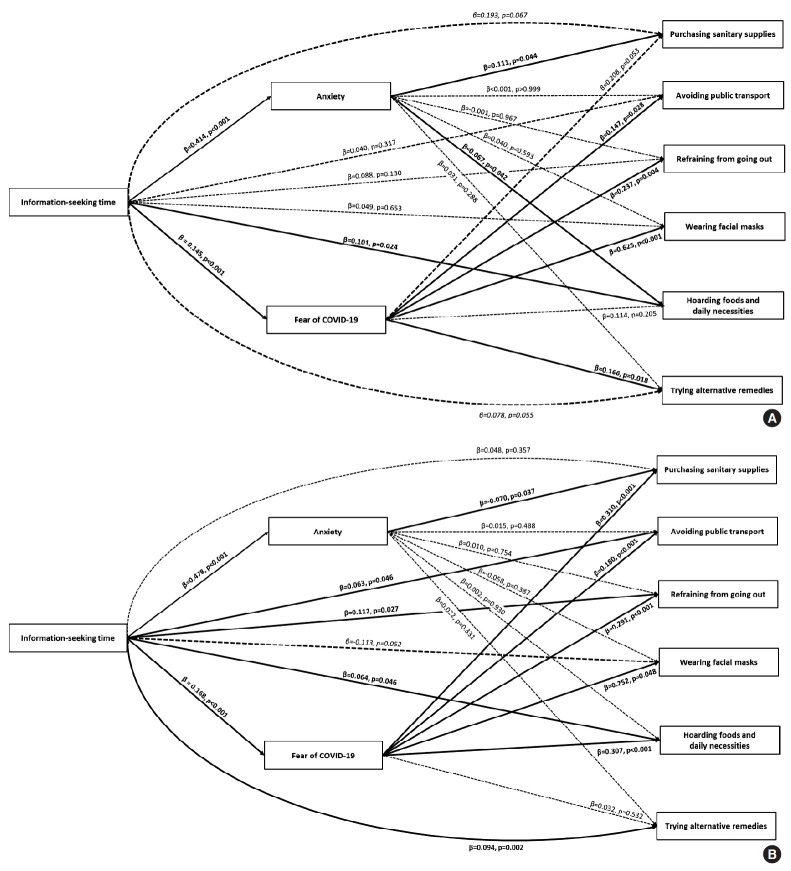
Conceptual diagram of the associations among information-seeking time, anxiety and fear of coronavirus disease 2019 (COVID-19), and prevention behaviours in men (A) and women (B).

Time spent seeking information was positively associated with anxiety (men: β=0.414, p<0.001; women: β=0.478, p<0.001) and fear (men: β=0.145, p<0.001; women: β=0.168, p<0.001). For men, anxiety was positively associated with purchasing sanitary supplies (OR per hour, 1.11; 95% CI, 1.00 to 1.23) and hoarding (OR per hour, 1.07; 95% CI, 1.00 to 1.14). No direct association between anxiety and prevention behaviours was found in women. Fear of COVID-19 was an important determinant of prevention behaviours for both men and women (Table 2 and Figure 3).

Sources of information about COVID-19 were found to be related to prevention behaviours. Exposure to Internet media showed a stronger association with prevention behaviours among women than among men (e.g., hoarding food and daily necessities [men: OR, 1.26; 95% CI, 0.78 to 2.02; women: OR, 2.40; 95% CI, 1.66 to 3.47]). Participants who received information from official institutions were more likely to wear face masks (men: OR, 3.01; 95% CI, 1.16 to 7.82; women: OR, 2.50; 95% CI, 1.22 to 5.11), refrain from going out (women: OR, 1.39; 95% CI, 1.01 to 1.90), purchase sanitary supplies (women: OR, 1.94; 95% CI, 1.29 to 2.90), and avoid public transportation (men: OR, 1.84; 95% CI, 1.30 to 2.61). In contrast, participants who relied on information from nonprofessional acquaintances were more likely to try unvalidated alternative remedies for COVID-19 prevention (Table 2).

### Mediating effects of anxiety and fear

In men, anxiety was found to have a mediating effect for the association between information-seeking behaviours and purchasing sanitary supplies and hoarding food and daily necessities (Table 3). In women, however, anxiety was not found to have a mediating effect for any prevention behaviours. The effects of time spent seeking information on prevention behaviours were partially mediated by fear of COVID-19. In men, the indirect effects of the fear of COVID-19 were positively associated with wearing face masks, purchasing sanitary supplies, avoiding public transportation, and trying folk remedies. In women, the fear of COVID-19 mediated the associations between time spent seeking information and prevention behaviours, including refraining from going out, purchasing sanitary supplies, avoiding public transportation, and hoarding food and daily necessities (Table 3).

Fear of COVID-19 mediated the associations between obtaining information from official institutions and prevention behaviours in women, including purchasing sanitary supplies, hoarding food and daily necessities, refraining from going out, wearing face masks, and avoiding public transportation. The fear of COVID-19 also mediated the associations between obtaining information from non-expert acquaintances and several prevention behaviours, including purchasing sanitary supplies, hoarding food and daily necessities, refraining from going out, and avoiding public transportation (Supplementary Material 1).

## DISCUSSION

Information-seeking patterns during the COVID-19 pandemic, including time spent seeking information and the sources of information, were associated with the adoption of several adaptive behaviours. In particular, spending more time researching information related to COVID-19 was found to increase one’s likelihood of adopting preventative behaviours against COVID-19. It is also noteworthy that, among the psychiatric traits, anxiety and fear rather than depressive symptoms and PTSS were the most influential mediators in the associations between informationseeking behaviours and behavioural adaptations related to COVID-19.

During an infectious disease pandemic, it has been found that behavioural changes, including social distancing, wearing face masks, and washing hands, are consequential enough to alter the patterns of infectious disease transmission and, ultimately, stop the disease from spreading further [38]. As such, it is important to identify and examine the determinants that affect prevention behaviours and prevention measures among the public. Since studies of the COVID-19 pandemic are still scarce, we aimed to provide data for understanding the role of information-seeking behaviours and general anxiety and fear as determinants of prevention behaviours.

The results of our study indicated that information-seeking patterns during the COVID-19 pandemic, including time spent seeking information and sources of information, were associated with the adoption of prevention behaviours recommended by government officials. It is important to note, however, that time spent seeking information also positively correlated with hoarding and trying unvalidated alternative remedies. A recent study from the United States showed that paying attention to information about COVID-19 was correlated with hoarding toilet paper and avoiding crowded spaces [39]. This suggests that, while individuals trying to gather information during a pandemic may be more likely to take protective actions, those actions are not necessarily limited to those recommended by authorities. Although hoarding is a common and understandable reaction of individuals living through difficult times, allowing others access to daily necessities is usually more beneficial for the community [40]. Trying unvalidated alternative remedies has also been a common phenomenon during the COVID-19 pandemic. Our results suggest that, although information-seeking is usually beneficial for the promotion of prevention behaviours, it might also be associated with non-beneficial behaviours.

We also found that sources of information were an important factor determining patterns of prevention behaviours. A previous study conducted in Germany found that specific sources of information affected compliance with the influenza A (H1N1) vaccination [7], suggesting that the impact of information-seeking is qualitatively different with respect to behavioural changes depending on the source. The results of our study also showed that several information sources promoted different prevention behaviours, some of which were beneficial and recommended by authorities, whereas others were not. Individuals who obtained information from official institutions were more likely to comply with recommended practices, such as purchasing sanitary supplies, wearing face masks, refraining from going out, and avoiding public transportation. This suggests that information provided by governmental organisations promotes compliance with recommended practices [41]. However, information received from non-expert acquaintances disproportionately influenced the participants to try folk remedies for the prevention of COVID-19, which, at best, have not been proven to be effective. These findings indicate that the source of information is as important as the quantity of information, and that, while some sources might provide useful and practical information for prevention, other sources might end up provoking negative behaviours in the community and inciting fear rather than promoting effective preventive measures [42].

In this study, anxiety and fear appeared to be important mediators through which information-seeking behaviours affected prevention behaviours. During the influenza A (H1N1) pandemic, several studies found that prevention behaviours adopted by the general public were influenced by public anxiety, perceptions of severity, and information-seeking behaviours [7,8,18], suggesting that anxiety among the general public could be used to predict the adoption of preventive measures. Health-related anxiety can promote the adoption of prevention behaviours since it can motivate the public to seek and implement appropriate remedies. Fear of the disease has also been shown to be a major determinant of prevention behaviours [20]. Theoretically, this phenomenon is explained by individuals’ motivation to decrease anxiety and fear, which results in behavioural adherence to preventive measures [43].

In this study, anxiety had a more prominent mediating effect for men, while a mediating effect of fear was observed in both genders. This suggests that, although the anxiety levels of men during the pandemic may be similar to those of women, men may be more likely to make behavioural modifications based on anxiety. Evidence from functional neuroimaging that supports this finding has shown that right prefrontal cortex activation, which is linked to the enhanced fight-or-flight reaction, is more apparent in men than in women [44]. These neurologic differences in reaction might explain men’s increased uptake of preventive measures in response to anxiety.

However, it should be noted that, although a certain level of anxiety and fear could help prevent the transmission of infectious diseases, they could also be ineffective or, under certain circumstances, harmful for disease prevention. When anxiety or fear exceeds the probable medical risk, its effects become counterproductive [45]. Therefore, while individuals should be aware of the state of the COVID-19 pandemic and adapt to its conditions, they should not be overwhelmed by panic and engage in behaviours that not only are not beneficial but are even potentially harmful to both individuals and their communities [46]. As such, effective risk communication is required to reduce negative emotions that could otherwise lead to decreased control over the public response to the pandemic [47].

This study is one of several to investigate the dynamics of behavioural responses to uncertainty and fear caused by the COVID-19 pandemic. Our results identified possible links between information-seeking and behavioural adaptations during the COVID-19 pandemic and the possible roles of anxiety and fear as mechanisms associated with such behaviours. Based on these findings, valid information should be provided by government officials and media in a controlled manner in order to control public fear and anxiety while fighting the spread of COVID-19 and reduce its impact on public health and well-being.

Despite this study’s strengths and its implications for public health policy, it also has several limitations. First, the present study might not represent the behaviours of all Koreans as most of the participants lived in Seoul and its surrounding areas. Second, only half of the invited participants responded to the online survey. Compared to the non-respondents, the respondents tended to be younger and more-educated and had fewer depressive symptoms at baseline (Supplemental Material 2). These differences between included and excluded participants could have led to selection bias. However, given that the difference in the mean Beck Depression Inventory-II score was only 1.66 points between respondents and non-respondents, the degree of bias was likely to be limited.

Finally, since the online survey was largely based on self-reported items, participants’ responses could have also been affected by response bias. The use of an objective measurement tool for assessing individuals’ responses to the COVID-19 pandemic would have been ideal, but the online nature of the survey limited us from adopting objective measurement techniques. Therefore, we had to rely on subjective responses instead, which is similar to the methods used in several previous studies [22-25,48-50]. More particularly, the single item used in this study to measure the fear of COVID-19 may not have accurately represented participants’ actual fear levels. Nevertheless, due to the lack of a pre-validated questionnaire for measuring the fear of emerging infectious diseases in respondents, several other studies have also measured fear using a single question [29-31]. Our study also used a single question, thus sharing the same limitation as previous studies.

Despite these limitations, our study highlights how information in the media can affect preventive strategies and behaviours among the general public and the importance of risk communication during a pandemic. Furthermore, our study demonstrated the psychological effects of information-seeking and resulting behavioural changes. Authorities, including public health experts, policymakers, and media, should provide valid, accurate, and thorough information and seek to mitigate public fear and anxiety in order to effectively undertake preventive strategies for controlling the ongoing COVID-19 pandemic in addition to future pandemics caused by other emerging infectious diseases.

## Figures and Tables

**Table 1.** Characteristics of the study population according to time spent seeking coronavirus disease 2019 (COVID-19)-related information

Characteristics		Men (n=673)			Women (n=1,228)		
		≤80 min/wk (n=354)	>80 min/wk (n=339)	p-value	≤80 min/wk (n=613)	>80 min/wk (n=615)	p-value
Age (yr)		50.46±10.22	50.85±9.76	0.620	50.65±9.19	51.23±8.69	0.253
Household income, percentile (10^6^ KRW/yr)				0.524			0.985
	<25 (<45.0)	74 (22.2)	66 (19.5)		168 (27.2)	171 (28.0)	
	25-50 (45.1-60.0)	126 (37.7)	124 (36.6)		214 (34.7)	206 (33.7)	
	50-75 (60.1-92.4)	61 (18.3)	59 (17.4)		108 (17.2)	107 (17.5)	
	≥75 (≥92.5)	73 (21.9)	90 (26.5)		127 (20.6)	127 (20.8)	
Education level (yr)				0.752			0.522
	Primary or lower (≤6)	5 (1.5)	3 (0.9)		20 (3.2)	23 (3.8)	
	Secondary (7-12)	105 (31.4)	105 (31.0)		298 (48.3)	276 (45.2)	
	Tertiary or higher (≥13)	224 (67.1)	231 (68.1)		299 (48.5)	312 (51.1)	
Charlson comorbidity index				0.692			0.204
	0	126 (37.7)	115 (33.9)		246 (39.9)	224 (36.7)	
	1	94 (28.1)	110 (32.4)		234 (37.9)	251 (41.1)	
	2	85 (25.4)	86 (25.4)		103 (16.7)	101 (16.5)	
	3	26 (7.8)	23 (6.8)		21 (3.4)	29 (4.7)	
	4	3 (0.9)	4 (1.2)		12 (1.9)	4 (0.6)	
	5	0 (0.0)	1 (0.3)		1 (0.2)	2 (0.3)	
Smoking status				0.915			0.334
	Non-smokers	94 (28.1)	91 (26.8)		575 (93.2)	574 (93.9)	
	Ex-smokers	151 (45.2)	154 (45.4)		28 (4.5)	19 (3.1)	
	Current smokers	89 (26.6)	94 (27.7)		14 (2.3)	18 (2.9)	
Drinking status				0.962			0.351
	Non-drinkers	30 (8.5)	30 (8.8)		174 (28.2)	161 (26.3)	
	Ex-drinkers	23 (6.5)	22 (6.5)		18 (2.9)	28 (4.6)	
	Light drinkers (men: ≤14 servings/wk; women: ≤7 servings/wk)	111 (31.4)	111 (32.7)		160 (25.9)	170 (27.8)	
	Heavy drinkers (men: >14 servings/wk; women: >7 servings/wk)	190 (53.7)	176 (51.9)		265 (42.9)	252 (41.2)	
Psychological health status							
	Baseline depression measured by BDI-II	8.17±6.2	8.15±6.14	0.970	9.71±6.32	9.69±6.4	0.944
	Depressive symptoms during the pandemic measured by PHQ-9	11.16±3.1	12.51±4.22	<0.001	10.99±3.08	12.36±4.2	<0.001
	Anxiety measured by GAD-7	3.29±4.1	5.32±4.71	<0.001	3.23±3.83	5.11±4.7	<0.001
	Posttraumatic stress symptoms measured by PCL-5	7.94±7.0	12.67±11.52	<0.001	8.22±9.21	12.45±11.1	<0.001
Source of information							
	Mass media (including Internet-based newspapers)	310 (92.8)	329 (97.0)	0.020	571 (92.5)	591 (96.7)	0.002
	Government organisations (including webpages, posters, and leaflets)	169 (47.7)	201 (59.3)	0.002	313 (47.0)	362 (59.2)	<0.001
	Medical personnel or institutions (including webpages)	61 (18.3)	95 (28.0)	0.004	105 (17.0)	144 (23.6)	0.005
	Portal sites and Internet media (including YouTube)	218 (65.3)	222 (65.5)	>0.999	392 (63.5)	438 (71.7)	0.003
	Other non-expert acquaintances	81 (24.2)	106 (31.3)	0.052	166 (26.9)	216 (35.3)	0.002
Prevention behaviours recommended by government officials							
	Wearing face masks	313 (93.7)	328 (96.8)	0.094	590 (95.6)	593 (97.0)	0.237
	Purchasing sanitary supplies	288 (86.2)	315 (92.9)	0.007	534 (86.5)	558 (91.3)	0.010
	Refraining from going out	246 (73.6)	285 (84.1)	0.001	450 (72.9)	534 (87.4)	<0.001
	Avoiding public transportation	174 (52.1)	208 (54.4)	0.019	300 (48.6)	357 (58.4)	<0.001
Prevention behaviours not recommended by government officials							
	Trying unvalidated alternative remedies	109 (32.6)	142 (56.6)	0.016	175 (28.4)	257 (42.1)	<0.001
	Hoarding foods and daily necessities	43 (12.9)	80 (23.6)	0.001	97 (15.7)	155 (25.4)	<0.001

Values are presented as mean±standard deviation or number (%). KRW, Korean won; BDI-II, Beck Depression Inventory-II; PHQ-9, Patient Health Questionnaire-9; GAD-7, Generalized Anxiety Disorder-7; PCL-5, Post-traumatic Stress Disorder Checklist-5.

**Table 2.** Associations between information-seeking behaviours, anxiety, fear, and the adoption of prevention behaviours^1^

Variables			Recommended by government officials				Not recommended by government officials	
			Wearing face masks	Purchasing sanitary supplies	Refraining from going out	Avoiding public transportation	Trying unvalidated alternative remedies	Hoarding food and daily necessities
Men (n=673)								
	Participants who adopted the behaviour, n (%)		641 (95.2)	603 (89.6)	531 (78.9)	382 (56.8)	251 (37.3)	123 (18.3)
	Time spent seeking COVID-19-related information		1.05 (0.85, 1.31)	1.21 (0.99, 1.49)	1.09 (0.97, 1.22)	1.05 (0.97, 1.13)	1.08 (1.00, 1.17)	1.12 (1.03, 1.22)
	Source of information							
		Mass media (including Internet-based newspapers)	3.19 (0.87, 11.76)	1.65 (0.61, 4.45)	1.41 (0.62, 3.21)	2.13 (1.00, 4.54)	1.21 (0.54, 2.73)	1.13 (0.42, 3.05)
		Government organisations (including webpages, posters, and leaflets)	2.32 (0.91, 5.92)	1.35 (0.76, 2.40)	1.25 (0.82, 1.92)	1.99 (1.39, 2.85)	1.10 (0.76, 1.61)	1.10 (0.68, 1.78)
		Medical personnel or medical institutions (including webpages)	3.70 (0.74, 18.46)	1.25 (0.60, 2.63)	1.72 (0.98, 3.01)	1.03 (0.68, 1.57)	0.95 (0.62, 1.45)	2.18 (1.33, 3.58)
		Other Internet-based media (including search engines and YouTube)	1.74 (0.78, 3.91)	1.46 (0.85, 2.52)	1.45 (0.97, 2.17)	0.98 (0.69, 1.39)	1.32 (0.92, 1.91)	1.20 (0.74, 1.92)
		Other non-expert acquaintances	4.45 (0.96, 20.64)	1.92 (0.95, 3.91)	1.12 (0.70, 1.80)	0.78 (0.54, 1.13)	1.85 (1.27, 2.70)	1.08 (0.67, 1.73)
	Depression measured by PHQ-9		1.04 (0.89, 1.20)	0.94 (0.86, 1.03)	1.00 (0.94, 1.07)	0.98 (0.93, 1.04)	0.85 (0.90, 1.01)	1.06 (0.99, 1.13)
	Anxiety measured by GAD-7		0.99 (0.86, 1.14)	1.11 (1.00, 1.24)	0.99 (0.92, 1.06)	1.00 (0.95, 1.06)	1.03 (0.97, 1.09)	1.07 (1.00, 1.15)
	Post-traumatic stress symptoms measured by PCL-5		0.96 (0.90, 1.02)	0.97 (0.93, 1.02)	1.00 (0.97, 1.03)	1.00 (0.97, 1.03)	1.01 (0.99, 1.04)	0.97 (0.94, 1.00)
	Fear of COVID-19		1.86 (1.32, 2.63)	1.24 (1.00, 1.53)	1.27 (1.08, 1.50)	1.14 (1.00, 1.30)	1.18 (1.03, 1.35)	1.08 (0.91, 1.29)
Women (n=1,228)								
	Participants who adopted the behaviour, n (%)		1,183 (96.3)	1,092 (88.9)	984 (80.1)	657 (53.5)	432 (35.2)	252 (20.5)
	Time spent seeking COVID-19-related information		0.90 (0.80, 1.00)	1.05 (0.95, 1.16)	1.12 (1.01, 1.24)	1.07 (1.00, 1.13)	1.09 (1.03, 1.16)	1.06 (1.00, 1.13)
	Source of information							
		Mass media (including Internet-based newspapers)	2.01 (0.63, 6.41)	1.79 (0.87, 3.69)	0.72 (0.36, 1.45)	1.61 (0.95, 2.72)	1.29 (0.72, 2.30)	1.08 (0.55, 2.10)
		Government organisations (including webpages, posters, and leaflets)	2.10 (1.02, 4.30)	2.04 (1.35, 3.07)	1.46 (1.06, 2.01)	1.34 (1.04, 1.73)	1.32 (1.01, 1.73)	0.81 (0.58, 1.12)
		Medical personnel or institutions (including webpages)	1.14 (0.41, 3.21)	1.36 (0.75, 2.48)	1.58 (1.01, 2.45)	0.92 (0.68, 1.26)	1.14 (0.83, 1.56)	1.63 (1.14, 2.34)
		Portal sites and Internet media (including YouTube)	3.09 (1.59, 6.03)	2.26 (1.54, 3.33)	1.49 (1.09, 2.04)	1.60 (1.23, 2.07)	1.11 (0.85, 1.46)	2.37 (1.65, 3.43)
		Other non-expert acquaintances	2.35 (0.95, 5.83)	1.07 (0.69, 1.65)	0.96 (0.68, 1.35)	0.94 (0.72, 1.22)	1.73 (1.32, 2.25)	0.99 (0.71, 1.37)
	Depression measured by PHQ-9		0.94 (0.83, 1.07)	0.98 (0.92, 1.06)	1.00 (0.94, 1.06)	0.99 (0.94, 1.03)	0.98 (0.93, 1.02)	1.02 (0.97, 1.07)
	Anxiety measured by GAD-7		1.12 (0.97, 1.30)	0.93 (0.87, 1.00)	1.01 (0.95, 1.07)	1.02 (0.97, 1.06)	1.02 (0.98, 1.07)	1.00 (0.95, 1.06)
	Posttraumatic stress symptoms measured by PCL-5		1.00 (0.95, 1.07)	1.01 (0.98, 1.04)	1.03 (1.00, 1.05)	0.99 (0.97, 1.01)	1.02 (1.00, 1.04)	1.01 (0.99, 1.03)
	Fear of COVID-19		1.24 (0.96, 1.60)	1.36 (1.17, 1.58)	1.35 (1.20, 1.52)	1.19 (1.08, 1.31)	1.03 (0.93, 1.14)	1.34 (1.19, 1.52)

Values are presented as odds ratio (95% confidence interval). COVID-19, coronavirus disease 2019; KRW, Korean won; BDI-II, Beck Depression Inventory-II; PHQ-9, Patient Health Questionnaire; GAD-7, Generalized Anxiety Disorder-7; PCL-5, Post-traumatic Stress Disorder Checklist-5.

1 All models were adjusted for age, household income, education level, drinking status, smoking status, comorbidities, baseline depressive symptoms, size of social network, and mean network closeness.

**Table 3.** Direct and indirect effects of time spent seeking information on prevention behaviours

Time spent seeking information			Implementing prevention behaviours^1^					
			Recommended by government officials				Not recommended by government officials	
			Wearing face masks	Refraining from going out	Purchasing sanitary supplies	Avoiding public transportation	Hoarding food and daily necessities	Trying alternative remedies
Men (n=673)								
	Direct effect		0.103 (-0.147, 0.353)	0.102 (-0.017, 0.220)	0.231 (0.018, 0.444)	0.048 (-0.031, 0.128)	0.100 (0.015, 0.185)	0.079 (0.001, 0.156)
	Indirect effect							
		Depressive symptoms	0.011 (-0.024, 0.062)	-0.001 (-0.021, 0.020)	-0.017 (-0.045, 0.003)	-0.007 (-0.026, 0.008)	0.012 (-0.005, 0.034)	-0.013 (-0.035, 0.002)
		Anxiety	-0.019 (-0.085, 0.056)	-0.006 (-0.038, 0.028)	0.038 (0.002, 0.095)	0.001 (-0.023, 0.028)	0.029 (0.002, 0.068)	0.009 (-0.016, 0.036)
		Post-traumatic stress symptoms	-0.028 (-0.098, 0.023)	-0.002 (-0.034, 0.028)	-0.013 (-0.052, 0.021)	0.000 (-0.024, 0.023)	-0.019 (-0.056, 0.005)	0.011 (-0.011, 0.040)
		Fear of COVID-19	0.085 (0.031, 0.184)	0.034 (0.009, 0.068)	0.033 (0.003, 0.074)	0.020 (0.000, 0.044)	0.016 (-0.008, 0.047)	0.024 (0.004, 0.053)
Women (n=1,228)								
	Direct effect		-0.099 (-0.212, 0.014)	0.113 (0.013, 0.213)	0.063 (-0.044, 0.171)	0.062 (0.002, 0.122)	0.059 (-0.002, 0.120)	0.094 (0.036, 0.152)
	Indirect effect							
		Depressive symptoms	-0.022 (-0.079, 0.028)	-0.002 (-0.030, 0.027)	-0.004 (-0.031, 0.023)	-0.003 (-0.021, 0.014)	0.011 (-0.009, 0.032)	-0.012 (-0.034, 0.004)
		Anxiety	0.055 (-0.032, 0.186)	0.001 (-0.030, 0.037)	-0.036 (-0.074, -0.002)	0.003 (-0.018, 0.025)	-0.002 (-0.027, 0.020)	0.010 (-0.012, 0.033)
		Post-traumatic stress symptoms	0.005 (-0.076, 0.101)	0.024 (-0.004, 0.059)	0.006 (-0.022, 0.041)	-0.009 (-0.032, 0.010)	0.008 (-0.014, 0.032)	0.018 (-0.011, 0.042)
		Fear of COVID-19	0.043 (-0.008, 0.103)	0.052 (0.030, 0.080)	0.056 (0.030, 0.091)	0.031 (0.015, 0.051)	0.051 (0.029, 0.792)	0.009 (-0.008, 0.027)

Values are presented as effect size (95% confidence interval). COVID-19, coronavirus disease 2019.

1 All effect sizes and their confidence intervals were estimated using the PROCESS macro in SAS by Andrew F. Hayes, conducting 5,000 simulations each for bootstrapping. All models were adjusted for age, household income, education level, drinking status, smoking status, comorbidities, baseline depressive symptoms, size of social network, and mean network closeness.

## SUPPLEMENTARY MATERIALS

Korean version is available at https://www.e-epih.org/.
