# Supplementary material for: The effects of information-seeking behaviours on prevention behaviours during the COVID-19 pandemic: the mediating effects of anxiety and fear in Korea
Source: Epidemiol Health. 2021 Oct 19;43:e2021085. doi: 10.4178/epih.e2021085 (PMC8863593; doi:10.4178/epih.e2021085)
Supplement: Supplementary file 1 [file epih-43-e2021085-suppl1.docx]

**Supplemental Material 1. Direct and indirect effects of information sources on preventive behaviors*.**

|  | **Recommended by government, ES (95% CI)** | | | | | | | **Not recommended by government, ES (95% CI)** | | | | |
| --- | --- | --- | --- | --- | --- | --- | --- | --- | --- | --- | --- | --- |
|  |  |  | |  | |  | | |  | |  | |
|  | **Wearing facial masks** | | **Purchasing sanitary supplies** | | **Refraining from going outside** | | **Avoiding public transport** | | | **Hoarding foods and daily necessities** | | **Trying folk remedies for COVID-19** |
| **Men (N=673)** | | | | | | | | | | | | |
|  | | | | | | | | | | | | |
| **Mass media (including internet-based newspapers)** |  | |  | |  | |  | | |  | |  |
| Direct effect | 1.091 (-0.068, 2.249) | | 0.837 (-0.057, 1.729) | | 0.416 (-0.358, 1.191) | | 0.882 (0.156, 1.608) | | | 0.062 (-0.862, 0.986) | | 0.384 (-0.389, 1.157) |
| Indirect effect - depressive symptoms | -0.020 (-0.215, 0.104) | | 0.047 (-0.048, 0.187) | | -0.000 (-0.096, 0.078) | | 0.019 (-0.036, 0.110) | | | -0.036 (-0.155, 0.038) | | 0.034 (-0.038, 0.153) |
| Indirect effect - anxiety | 0.003 (-0.137, 0.166) | | -0.032 (-0.238, 0.139) | | 0.001 (-0.071, 0.074) | | -0.001 (-0.055, 0.057) | | | -0.008 (-0.157, 0.128) | | -0.002 (-0.079, 0.060) |
| Indirect effect - posttraumatic symptoms | 0.036 (-0.090, 0.262) | | 0.022 (-0.080, 0.176) | | 0.000 (-0.091, 0.115) | | -0.002 (-0.076, 0.075) | | | 0.035 (-0.075, 0.208) | | -0.021 (-0.150, 0.052) |
| Indirect effect - fear of COVID-19 | 0.083 (-0.216, 0.436) | | 0.027 (-0.104, 0.181) | | 0.036 (-0.090, 0.183) | | 0.019 (-0.053, 0.105) | | | 0.019 (-0.055, 0.119) | | 0.027 (-0.070, 0.139) |
| **Government organizations (including webpages, posters and leaflets)** |  | |  | |  | |  | | |  | |  |
| Direct effect | **2.045 (1.190, 2.900)** | | **0.962 (0.457, 1.468)** | | **0.691 (0.307, 1.074)** | | **0.748 (0.428, 1.067)** | | | 0.276 (-0.139, 0.690) | | **0.338 (0.007, 0.669)** |
| Indirect effect - depressive symptoms | -0.007 (-0.073, 0.041) | | 0.005 (-0.024, 0.052) | | -0.002 (-0.034, 0.026) | | 0.033 (-0.016, 0.037) | | | -0.008 (-0.049, 0.029) | | 0.007 (-0.019, 0.055) |
| Indirect effect - anxiety | 0.005 (-0.092, 0.100) | | -0.001 (-0.042, 0.035) | | 0.002 (-0.043, 0.046) | | 0.000 (-0.026, 0.025) | | | -0.004 (-0.068, 0.060) | | -0.001 (-0.030, 0.027) |
| Indirect effect - posttraumatic symptoms | -0.020 (-0.120, 0.048) | | -0.008 (-0.063, 0.033) | | 0.001 (-0.030, 0.047) | | -0.001 (-0.034, 0.027) | | | -0.014 (-0.073, 0.035) | | 0.008 (-0.019, 0.053) |
| Indirect effect - fear of COVID-19 | 0.126 (-0.112, 0.400) | | 0.062 (-0.051, 0.201) | | 0.049 (-0.040, 0.151) | | 0.028 (-0.025, 0.089) | | | 0.022 (-0.021, 0.078) | | 0.030 (-0.026, 0.096) |
| **Medical personnel or medical institutes (including webpages)** |  | |  | |  | |  | | |  | |  |
| Direct effect | **1.493 (0.035, 2.952)** | | 0.471 (-0.212, 1.155) | | **0.713 (0.195, 1.232)** | | 0.251 (-0.121, 0.622) | | | **0.841 (0.402, 1.281)** | | 0.182 (-0.194, 0.559) |
| Indirect effect - depressive symptoms | -0.002 (-0.059, 0.046) | | 0.006 (-0.045, 0.059) | | 0.000 (-0.027, 0.028) | | 0.003 (-0.022, 0.037) | | | -0.005 (-0.049, 0.040) | | 0.005 (-0.034, 0.047) |
| Indirect effect - anxiety | -0.006 (-0.081, 0.082) | | 0.031 (-0.047, 0.143) | | -0.004 (-0.046, 0.038) | | 0.002 (-0.023, 0.043) | | | 0.023 (-0.033, 0.105) | | 0.007 (-0.023, 0.046) |
| Indirect effect - posttraumatic symptoms | -0.029 (-0.154, 0.032) | | -0.015 (-0.084, 0.041) | | -0.002 (-0.055, 0.049) | | -0.001 (-0.038, 0.037) | | | -0.025 (-0.107, 0.020) | | 0.013 (-0.019, 0.067) |
| Indirect effect - fear of COVID-19 | 0.070 (-0.075, 0.265) | | 0.032 (-0.035, 0.116) | | 0.031 (-0.032, 0.114) | | 0.017 (-0.019, 0.069) | | | 0.016 (-0.021, 0.074) | | 0.023 (-0.025, 0.083) |
| **Other internet-based media (including search engines and YouTube)** |  | |  | |  | |  | | |  | |  |
| Direct effect | 0.698 (-0.033, 1.430) | | 0.417 (-0.101, 0.936) | | **0.572 (0.188, 0.957)** | | 0.094 (-0.231, 0.420) | | | 0.393 (-0.050, 0.837) | | **0.348 (0.005, 0.692)** |
| Indirect effect - depressive symptoms | 0.007 (-0.071, 0.098) | | -0.034 (-0.105, 0.012) | | -0.003 (-0.052, 0.039) | | -0.014 (-0.059, 0.016) | | | 0.022 (-0.013, 0.077) | | -0.025 (-0.074, 0.009) |
| Indirect effect - anxiety | -0.012 (-0.094, 0.080) | | 0.036 (-0.036, 0.151) | | -0.006 (-0.050, 0.038) | | 0.003 (-0.026, 0.040) | | | 0.027 (-0.028, 0.104) | | 0.009 (-0.023, 0.050) |
| Indirect effect - posttraumatic symptoms | -0.016 (-0.099, 0.041) | | -0.009 (-0.074, 0.034) | | 0.000 (-0.040, 0.042) | | 0.000 (-0.027, 0.033) | | | -0.015 (-0.075, 0.034) | | 0.009 (-0.019, 0.058) |
| Indirect effect - fear of COVID-19 | 0.052 (-0.085, 0.225) | | 0.023 (-0.040, 0.100) | | 0.022 (-0.036, 0.093) | | 0.012, (-0.022, 0.055) | | | 0.012 (-0.021, 0.059) | | 0.017 (-0.026, 0.071) |
| **Other non-expert acquaintances** |  | |  | |  | |  | | |  | |  |
| Direct effect | **1.722 (0.266, 3.177)** | | **0.760 (0.082, 1.439)** | | 0.363 (-0.084, 0.811) | | -0.043 (-0.389, 0.302) | | | 0.294 (-0.142, 0.729) | | **0.658 (0.304, 1.012)** |
| Indirect effect - depressive symptoms | -0.001 (-0.52, 0.045) | | 0.003 (-0.050, 0.058) | | 0.000 (-0.025, 0.028) | | 0.001 (-0.028, 0.034) | | | -0.002 (-0.043, 0.041) | | 0.002 (-0.035, 0.043) |
| Indirect effect - anxiety | -0.003 (-0.068, 0.078) | | 0.019 (-0.068, 0.132) | | -0.002 (-0.039, 0.036) | | 0.001 (-0.023, 0.036) | | | 0.014 (-0.044, 0.090) | | 0.005 (-0.028, 0.043) |
| Indirect effect - posttraumatic symptoms | -0.024 (-0.136, 0.040) | | -0.013 (-0.083, 0.035) | | -0.001 (-0.043, 0.051) | | 0.000 (-0.031, 0.038) | | | -0.019 (-0.090, 0.028) | | 0.009 (-0.022, 0.059) |
| Indirect effect - fear of COVID-19 | 0.103 (-0.025, 0.288) | | 0.047 (-0.011, 0.139) | | 0.046 (-0.010, 0.127) | | 0.026 (-0.007, 0.080) | | | 0.024 (-0.011, 0.087) | | 0.034 (-0.008, 0.097) |
|  |  | |  | |  | |  | | |  | |  |
|  |  | |  | |  | |  | | |  | |  |
|  |  | |  | |  | |  | | |  | |  |
|  |  | |  | |  | |  | | |  | |  |
|  |  | |  | |  | |  | | |  | |  |
|  |  | |  | |  | |  | | |  | |  |
|  |  | |  | |  | |  | | |  | |  |
| **Women (N=1228)** | | | | | | | | | | | | |
|  | | | | | | | | | | | | |
| **Mass media (including internet-based newspapers)** |  | |  | |  | |  | | |  | |  |
| Direct effect | 1.196 (-0.806, 3.198) | | 0.757 (-0.173, 1.686) | | **1.062 (0.265, 1.858)** | | 0.061 (-0.384, 0.506) | | | **0.684 (0.192, 1.176)** | | 0.307 (-0.142, 0.757) |
| Indirect effect - depressive symptoms | 0.015 (-0.056, 0.104) | | 0.001 (-0.033, 0.035) | | -0.001 (-0.038, 0.036) | | 0.001 (-0.021, 0.022) | | | -0.010 (-0.056, 0.023) | | 0.006 (-0.018, 0.041) |
| Indirect effect - anxiety | 0.037 (-0.114, 0.297) | | -0.027 (-0.129, 0.057) | | 0.002 (-0.042, 0.053) | | 0.004 (-0.025, 0.043) | | | -0.001 (-0.034, 0.035) | | 0.009 (-0.020, 0.060) |
| Indirect effect - posttraumatic symptoms | 0.006 (-0.130, 0.172) | | 0.004 (-0.054, 0.054) | | 0.021 (-0.044, 0.120) | | -0.008 (-0.057, 0.026) | | | 0.004 (-0.034, 0.045) | | 0.016 (-0.034, 0.084) |
| Indirect effect - fear of COVID-19 | 0.040 (-0.035, 0.166) | | 0.059 (-0.045, 0.185) | | 0.056 (-0.043, 0.167) | | 0.034 (-0.027, 0.103) | | | 0.055 (-0.041, 0.166) | | 0.012 (-0.011, 0.052) |
| **Government organizations (including webpages, posters and leaflets)** |  | |  | |  | |  | | |  | |  |
| Direct effect | **1.781 (1.163 - 2.399)** | | **1.241 (0.845, 1.584)** | | **0.744 (0.457, 1.031)** | | 0.431 (0.201, 0.660) | | | 0.161 (-0.127, 0.449) | | **0.511 (0.270, 0.753)** |
| Indirect effect - depressive symptoms | 0.009 (-0.019, 0.054) | | 0.022 (-0.015, 0.023) | | 0.000 (-0.020, 0.015) | | 0.001 (-0.011, 0.015) | | | -0.006 (-0.031, 0.011) | | 0.004 (-0.008, 0.024) |
| Indirect effect - anxiety | -0.004 (-0.058, 0.041) | | -0.006 (-0.067, 0.051) | | -0.001 (-0.021, 0.020) | | 0.000 (-0.012, 0.014) | | | -0.000 (-0.013, 0.015) | | 0.009 (-0.012, 0.018) |
| Indirect effect - posttraumatic symptoms | -0.005 (-0.050, 0.042) | | 0.011 (-0.022, 0.029) | | 0.007 (-0.013, 0.044) | | -0.007 (-0.033, 0.010) | | | 0.003 (-0.017, 0.024) | | 0.008 (-0.009, 0.039) |
| Indirect effect - fear of COVID-19 | **0.180 (0.067, 0.343)** | | **0.133 (0.053, 0.239)** | | **0.118 (0.045, 0.210)** | | **0.068 (0.025, 0.124)** | | | **0.092 (0.034, 0.166)** | | **0.030 (0.002, 0.069)** |
| **Medical personnel or medical institutes (including webpages)** |  | |  | |  | |  | | |  | |  |
| Direct effect | 0.677 (-0.270, 1.624) | | **0.793 (0.232, 1.353)** | | **0.592 (0.180, 1.004)** | | 0.096 (-0.188, 0.380) | | | **0.531 (0.203, 0.859)** | | 0.389 (0.101, 0.677) |
| Indirect effect - depressive symptoms | -0.020 (-0.097, 0.033) | | -0.005 (-0.042, 0.023) | | 0.000 (-0.038, 0.031) | | -0.001 (-0.023, 0.020) | | | 0.010 (-0.011, 0.045) | | -0.009 (-0.039, 0.011) |
| Indirect effect - anxiety | 0.037 (-0.041, 0.215) | | -0.030 (-0.100, 0.017) | | 0.000 (-0.031, 0.037) | | 0.004 (-0.018, 0.032) | | | -0.001 (-0.026, 0.025) | | 0.009 (-0.011, 0.043) |
| Indirect effect - posttraumatic symptoms | 0.002 (-0.065, 0.079) | | 0.002 (-0.024, 0.040) | | 0.005 (-0.042, 0.059) | | -0.002 (-0.029, 0.018) | | | 0.002 (-0.018, 0.030) | | 0.004 (-0.028, 0.041) |
| Indirect effect - fear of COVID-19 | 0.046 (-0.013, 0.152) | | 0.066 (-0.004, 0.155) | | 0.064 (-0.004, 0.145) | | 0.039 (-0.003, 0.089) | | | 0.059 (-0.004, 0.131) | | 0.014 (-0.006, 0.043) |
| **Other internet-based media (including search engines and YouTube)** |  | |  | |  | |  | | |  | |  |
| Direct effect | **1.170 (0.561, 1.778)** | | **0.984 (0.619, 1.349)** | | 0.525 (0.226, 0.823) | | 0.461 (0.217, 0.704) | | | **0.851 (0.508, 1.194)** | | **0.259 (0.003, 0.516)** |
| Indirect effect - depressive symptoms | -0.022 (-0.089, 0.027) | | -0.008 (-0.042, 0.016) | | -0.001 (-0.034, 0.027) | | -0.003 (-0.023, 0.015) | | | 0.008 (-0.010, 0.036) | | -0.009 (-0.034, 0.007) |
| Indirect effect - anxiety | -0.017 (-0.124, 0.064) | | 0.011 (-0.028, 0.056) | | -0.002 (-0.029, 0.017) | | -0.002 (-0.023, 0.013) | | | -0.001 (-0.021, 0.015) | | -0.004 (-0.031, 0.013) |
| Indirect effect - posttraumatic symptoms | 0.001 (-0.051, 0.067) | | 0.001 (-0.018, 0.031) | | 0.004 (-0.030, 0.051) | | -0.002 (-0.026, 0.015) | | | 0.001 (-0.016, 0.023) | | 0.003 (-0.022, 0.036) |
| Indirect effect - fear of COVID-19 | 0.009 (-0.036, 0.074) | | 0.014 (-0.048, 0.080) | | 0.014 (-0.045, 0.075) | | 0.008 (-0.027, 0.046) | | | 0.013 (-0.042, 0.071) | | 0.003 (-0.012, 0.023) |
| **Other non-expert acquaintances** |  | |  | |  | |  | | |  | |  |
| Direct effect | **1.055 (0.178, 1.932)** | | 0.211 (-0.123, 0.703) | | 0.130 (-0.195, 0.454) | | 0.070 (-0.177, 0.318) | | | 0.161 (-0.140, 0.463) | | **0.626 (0.373, 0.880)** |
| Indirect effect - depressive symptoms | 0.000 (-0.044, 0.037) | | 0.000 (-0.019, 0.017) | | 0.000 (-0.021, 0.016) | | 0.000 (-0.012, 0.011) | | | 0.000 (-0.021, 0.022) | | 0.000 (-0.013, 0.016) |
| Indirect effect - anxiety | 0.052 (-0.052, 0.223) | | -0.041 (-0.110, 0.005) | | 0.002 (-0.037, 0.048) | | 0.006 (-0.020, 0.040) | | | 0.000 (-0.032, 0.031) | | 0.013 (-0.010, 0.050) |
| Indirect effect - posttraumatic symptoms | 0.009 (-0.137, 0.183) | | 0.011 (-0.043, 0.077) | | 0.040 (-0.006, 0.115) | | -0.016 (-0.061, 0.016) | | | 0.008 (-0.029, 0.053) | | 0.026 (-0.004, 0.075) |
| Indirect effect - fear of COVID-19 | 0.056 (-0.015, 0.168) | | **0.086 (0.022, 0.169)** | | **0.083 (0.023, 0.158)** | | **0.050 (0.012, 0.101)** | | | **0.077 (0.020, 0.147)** | | 0.016 (-0.007, 0.049) |

ES, effect size; CI, confidence interval;

*All effect sizes and their confidence intervals were estimated using 'Process' Macro by Andrew F. Hayes, with 5,000 simulations each for bootstrapping. All models are adjusted for age, household income, degree of education, alcohol consumption, cigarette smoking, comorbidities, baseline depressive symptoms, size of social network, and mean network closeness.
