# Supplementary material for: The effects of information-seeking behaviours on prevention behaviours during the COVID-19 pandemic: the mediating effects of anxiety and fear in Korea
Source: Epidemiol Health. 2021 Oct 19;43:e2021085. doi: 10.4178/epih.e2021085 (PMC8863593; doi:10.4178/epih.e2021085)
Supplement: Supplementary file 2 [file epih-43-e2021085-suppl2.docx]

**Supplemental Material 2. Differences in characteristics of included and excluded participants.**

|  | **Included (N=1901)** | **Excluded (N=2159)** | **p-value** |
| --- | --- | --- | --- |
| **Age, mean (SD)** | 50.84 (9.33) | 51.73 (8.36) | **0.003** |
| **Men, N (%)** | 673 (35.40) | 753 (34.88) | 0.751 |
| **Degree of education, N (%)** |  |  | **<0.001** |
| Primary or lower | 51 (2.68) | 179 (8.29) |  |
| Secondary | 784 (41.24) | 1151 (53.31) |  |
| Teritiary or higher | 1066 (56.08) | 828 (38.35) |  |
| N/A | 0 (0) | 1 (0.05) |  |
| **Baseline depression measured by BDI-II, mean (SD)** | 9.15 (6.35) | 10.48 (8.01) | **<0.001** |
| **Charlson Comorbidity Index, N (%)** |  |  | 0.055 |
| 0 | 711 (37.40) | 741 (34.32) |  |
| 1 | 689 (36.24) | 767 (35.53) |  |
| 2 | 375 (19.73) | 511 (23.67) |  |
| 3 | 99 (5.21) | 106 (4.91) |  |
| 4 | 23 (1.21) | 30 (1.39) |  |
| 5 | 4 (0.21) | 3 (0.14) |  |
| 6 | 0 (0) | 1 (0.05) |  |
| **Cigarette smoking, N (%)** |  |  | **<0.001** |
| Nonsmoker | 1334 (70.17) | 1446 (66.98) |  |
| Ex-smoker | 352 (18.52) | 372 (17.23) |  |
| Current smoker | 215 (11.31) | 341 (15.79) |  |
| **Alcohol consumption, N (%)** |  |  | 0.062 |
| Nondrinker | 395 (20.78) | 511 (23.67) |  |
| Ex-drinker | 90 (4.73) | 94 (4.35) |  |
| Light drinkers (Men: ≤14 servings/week; women: ≤7 servings/week) | 544 (28.62) | 640 (29.64) |  |
| Heavy drinkers (Men: >14 servings/week; women: >7 servings/week) | 872 (45.87) | 914 (42.33) |  |

SD, standard deviation; N/A, not available
